# Circulating miRNAs: cell–cell communication function?

**DOI:** 10.3389/fgene.2013.00119

**Published:** 2013-06-28

**Authors:** A. Turchinovich, T. R. Samatov, A. G. Tonevitsky, B. Burwinkel

**Affiliations:** ^1^Molecular Epidemiology Group, C080, German Cancer Research Center (DKFZ)Heidelberg, Germany; ^2^Division of Molecular Biology of Breast Cancer, Department of Gynecology and Obstetrics, University Women's Clinic, University HeidelbergHeidelberg, Germany; ^3^SRC BioclinicumMoscow, Russia; ^4^Institute of General Pathology and Pathophysiology, Russian Academy of Medical ScienceMoscow, Russia; ^5^M. V. Lomonosov Moscow State UniversityMoscow, Russia

**Keywords:** miRNA, biofluids, cell communication, argonaute proteins, exosomes, microvesicles, HDL, biomarkers

## Abstract

Nuclease resistant extracellular miRNAs have been found in all known biological fluids. The biological function of extracellular miRNAs remains questionable; however, strong evidence suggests that these miRNAs can be more than just byproducts of cellular activity. Some extracellular miRNA species might carry cell–cell signaling function during various physiological and pathological processes. In this review, we discuss the state-of-the-art in the field of intercellular miRNA transport and highlight current theories regarding the origin and the biological function of extracellular miRNAs.

## Introduction

MicroRNAs (miRNAs), a subclass of short non-coding RNAs, are expressed in all eukaryotic cell types and mediate posttranscriptional regulation of gene expression (Ambros, 2004; Bartel, 2004). The action of miRNAs is mediated by its binding to the 3′-untranslated region (3′UTR) of the target mRNAs and thus regulating targeted mRNAs stability and protein synthesis (Ambros, 2004; Bartel, 2004). There have been more than 2000 different human miRNA species discovered so far and this amount is increasing (http://www.mirbase.org/). In humans, endogenous miRNAs regulate at least 30% of genes (Lewis et al., 2005) and, thus, coordinate key cellular processes including proliferation, DNA repair, differentiation, metabolism, and apoptosis (Ambros, 2004; Bartel, 2004; Croce and Calin, 2005). Deregulation of certain miRNAs' expression in the cell was consistently observed during various pathologies including cancers (Lu et al., 2005). Every miRNA has a unique nucleotide sequence and unique expression pattern in certain cell type (Lu et al., 2005; Landgraf et al., 2007).

Several years ago, significant amounts of miRNA were detected in all biological fluids including blood plasma, urine, tears, breast milk, amniotic fluid, cerebrospinal fluid, saliva, and semen [reviewed in Turchinovich et al. (2012)]. These extracellular circulating miRNAs are surprisingly stable and survive unfavorable physiological conditions such as extreme variations in pH, boiling, multiple freeze thaw cycles, and extended storage. In contrast to miRNAs, common RNA species like mRNA, rRNA, and tRNA are degraded within several seconds after being placed in nuclease rich extracellular environment (Chen et al., 2008; Turchinovich et al., 2011). In their pioneering work Valadi et al. reported that cells in culture transport intracellular miRNAs into the extracellular environment by exosomes (Valadi et al., 2007). This finding was confirmed in many subsequent reports and mechanisms of miRNA transfer have been suggested. In this review, we discuss the state-of-the-art in the field of intercellular miRNA transport, and particularly the mechanisms involved in this process. We will highlight actual theories regarding the origin and the biological function of extracellular circulating miRNAs in body fluids.

## miRNA biogenesis and mode of action

**Figure 1:**
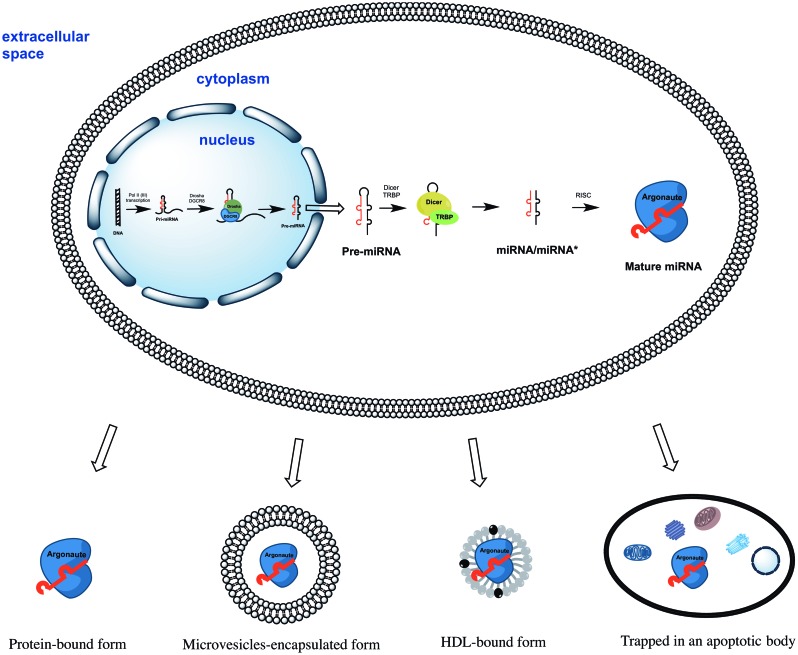
**The biogenesis of miRNAs starts in the cell nucleus with generation of primary miRNAs (pri-miRNAs) transcripts**. Pri-miRNAs are cleaved by the microprocessor complex Drosha/DGCR8 into shorter miRNA precursors (pre-miRNA). The later are transported to the cytoplasm and further cut by the endonuclease Dicer into ~22 nt miRNA/miRNA^*^ duplexes. Finally, one of the miRNA/miRNA^*^ strands is incorporated into a protein of the Argonaute family (AGO1, AGO2, AGO3, or AGO4). The mature miRNA strand eventually serves as the guide for RISC-mediated mRNA targeting resulting in either mRNA cleavage or translational interference. Extracellular miRNA can be either solely AGO protein-associated or additionally encapsulated into apoptotic bodies, microvesicles, and HDL particles.

All miRNAs are originally generated in the cell nucleus as long primary miRNAs (pri-miRNAs) transcripts containing 5′cap and a 3′polyA tail (Lee et al., 2004). The pri-miRNAs are further cleaved by a microprocessor complex consisting of Drosha and DGCR8 proteins into ~70 nt hairpin precursor miRNAs (pre-miRNAs) (Lee et al., 2003; Landthaler et al., 2004). On the next step pre-miRNAs are actively transported into the cytoplasm (Yi et al., 2003) where they cleaved into ~22 bp miRNA/miRNA^*^ duplexes by Dicer/TRBP enzyme complex (Zhang et al., 2002; Chendrimada et al., 2005). Finally, miRNA/miRNA^*^ duplexes separate leaving one of strands associated with an Argonaute (AGO) protein (Okamura et al., 2004; Ender and Meister, 2010). This AGO associated “mature” miRNA strand sequence-specifically binds to complementary mRNAs, promoting their decay and inhibiting translation (Figure 1). Surprisingly, some miRNAs activated both translation and steady state levels of target mRNAs during cell cycle arrest in quiescent mammalian cells and Xenopus oocytes (Vasudevan et al., 2007; Truesdell et al., 2012)—a mechanism which is yet to be explained.

Four human AGO proteins (AGO1, AGO2, AGO3, and AGO4) have been described so far; all of them can mediate both translation repression of mRNA on ribosomes and mRNA decay in P-bodies. However, only AGO2 is capable of directly cleaving mRNA in the cytoplasm (Hock and Meister, 2008). AGO carrying miRNA forms the RNA-induced silencing complex (RISC) by binding to GW182 via C-terminal domain of AGO protein (Lian et al., 2009; Braun et al., 2013). The RISC can be localized (1) diffusely in the cytoplasm; (2) in the dense cytoplasmic structures called GW- or P-bodies—the main localization for mRNAs which undergo decapping, deadenylation, and degradation (Kedersha et al., 2005). Importantly, only 1% of cytoplasmic AGO2 was found in P-bodies whereas the majority was diffusely distributed elsewhere in the cytoplasm (Leung and Sharp, 2013). When studying extracellular miRNA the researchers must take into account the particularities of miRNA biogenesis, its mode of action and localization in the cell. Importantly, neither mature miRNAs nor pre-miRNAs were ever found within the cells in non-protein bound forms.

## Extracellular miRNA

The pioneering observations that mature miRNAs are also present in cell-free blood plasma and serum was made in the year of 2008 by several independent research groups (Chen et al., 2008; Chim et al., 2008; Lawrie et al., 2008; Mitchell et al., 2008). Later, the existence of extracellular circulating miRNA in all other biological fluids was confirmed (Park et al., 2009; Hanke et al., 2010; Kosaka et al., 2010b; Weber et al., 2010). The mechanism which is responsible for the nuclease resistance of miRNA outside the cell remained enigmatic for quite a long period; however, the presence of miRNAs in the exosomes exported by cells in culture has been known before (Valadi et al., 2007). The theory that extracellular miRNA is protected by encapsulation into membrane-vesicles emerged after Hunter et al. detected miRNAs in peripheral blood microvesicles (Hunter et al., 2008). Together with the evidence that exchange of miRNA (and also mRNA) between cells can be accomplished through exosome-mediated transfer (Valadi et al., 2007) the finding of Hunter and co-authors led to a revolutionary hypothesis—the existence intercellular and inter-organ communication system in the body by means of microvesicles (MVs) encapsulated miRNAs.

In 2011, the assumption that only membrane-vesicles encapsulated miRNAs are present in biological fluids was challenged by two independent research groups who demonstrated that 90–99% of extracellular miRNA are MVs-free and associated with proteins of the AGO family both in blood plasma/serum and cell culture media (Arroyo et al., 2011; Turchinovich et al., 2011). The remarkable stability of AGO2 protein in protease rich environment elegantly explained the resistance of associated miRNAs in nucleases containing biological fluids (Turchinovich et al., 2011).

Since then, accumulated reports have consistently shown that extracellular miRNAs can be shielded from RNAse degradation by: (1) packaging in apoptotic bodies, shedding vesicles and exosomes; or (2) solely by complexing with AGO proteins (reviewed in Cortez et al., 2011; Chen et al., 2012; Turchinovich et al., 2012) (Figure 1). Some miRNA species were also found in purified fractions of high-density lipoprotein (HDL) from human plasma (Vickers et al., 2011). The existence of HDL-associated miRNAs in the blood circulation have been recently confirmed by Dimmeler's group (Wagner et al., 2013), however, the analysed HDL-miRNAs constituted only minor proportion the total circulating miRNAs. Finally, synthetic miRNA can be protected from the degradation by RNAses when mixed with purified nucleophosmin 1 (NPM1) protein *in vitro* (Wang et al., 2010). Although NPM1 was indeed exported by cells in culture together with miRNA, neither intracellular nor extracellular miRNA association with NPM1 has been found *in vivo* (Wang et al., 2010; Turchinovich et al., 2011).

## The theory of cell–cell communication via extracellular miRNA

The presence of miRNA in the extracellular environment ignited the hypotheses that cells selectively release miRNAs which mediate cell–cell signaling via paracrine or even endocrine routes (Valadi et al., 2007; Cortez et al., 2011; Chen et al., 2012). However, circulating miRNAs bound solely by AGO proteins are apparently non-specific remnants resulting from physiological activity of the cells and cell death (Turchinovich et al., 2011; Turchinovich and Burwinkel, 2012). Thus, both AGO2 protein and miRNAs remain stable for prolonged periods after the parental cells die. Furthermore, there are no indications of either active release of AGO-miRNA ribonucleoprotein complexes from cells or their uptake by recipient cells in mammals. The opinion that many extracellular miRNAs are released non-selectively after cell death also accords with the fact that upon toxicity in certain tissues the level of tissue-specific miRNAs in the blood increases (Laterza et al., 2009; Corsten et al., 2010; Lewis and Jopling, 2010; Zhang et al., 2010a; Pritchard et al., 2012).

At the same time, a number of independent research groups have demonstrated that extracellular miRNAs entrapped within apoptotic bodies and exosomes can be transferred to recipient cells, alter gene expression and mediate functional effects (Valadi et al., 2007; Skog et al., 2008; Kosaka et al., 2010a; Pegtel et al., 2010; Mittelbrunn et al., 2011; Montecalvo et al., 2012). Patterns of mRNAs in exosomes and their donor cells correlate poorly, suggesting specific sorting of miRNA “for export” (Valadi et al., 2007; Skog et al., 2008; Collino et al., 2010; Pigati et al., 2010; Mittelbrunn et al., 2011). The mechanism behind this sorting needs to be investigated in more detail, however, certain clues may lie within the fact that miRNAs, GW182 and AGO proteins co-localize in the compartments which are strongly linked with endosomes and multivesicular bodies (MVBs) (Gibbings et al., 2009). Because exosomes are formed in the MVB and also contain high levels of GW182, these observations may be important findings for the understanding of the loading of RNA into exosomes (Gibbings et al., 2009). It is feasible that AGO-bound miRNAs which reside in the MVBs become encapsulated randomly into the newly formed exosomes. The fact that different miRNAs might possess different decay kinetics could partially account for the fact that certain miRNAs were expressed at higher levels in extracellular MVs than in the parental cells (Bail et al., 2010; Krol et al., 2010). Another possible methodological bias which has to be addressed when comparing extracellular versus intracellular miRNA profiles include preferential loss of certain miRNAs during extraction from samples with very low RNA content (e.g., extracellular fluids) (Kim et al., 2012). Nevertheless, there is mounting evidence that cells selectively package certain miRNAs into MVs and actively secrete them. However the exact mechanisms of vesicular miRNAs sorting and secretion are yet to be discovered.

Collino and co-authors have demonstrated that MVs exported by human bone marrow derived mesenchymal stem cells (MSCs) and liver resident stem cells (HLSCs) indeed contained both miRNAs and AGO2 protein (Collino et al., 2010). Furthermore, selected patterns of miRNAs in MVs suggested their specific compartmentalization. Bioinformatics analysis revealed that MV-expressed miRNAs could be involved in organ development, cell survival, cell differentiation, and regulation of the immune system. The authors further showed that pre-treatment with the inhibitor of actin polymerization cytochalasin B significantly reduced the release of MVs from both MSCs and HLSCs (Collino et al., 2010).

At the same time several research groups have further demonstrated that exosomal miRNA is released via ceramide-dependent secretory pathway which is controlled by the enzyme of ceramide biosynthesis neutral sphingomyelinase (nSMase) (Kosaka et al., 2010a; Kogure et al., 2011; Mittelbrunn et al., 2011). nSMase mediates hydrolysis of sphingomyelin to form ceramide and is indispensable for budding of intracellular vesicles into the MVB (Trajkovic et al., 2008). Inhibition of nSMase2 with the small molecule compound GW4869 and the appropriate siRNA decreased both exosomes and miRNA secretion (Kosaka et al., 2010a). Consistently, ectopic overexpression of nSMase2 resulted in higher amounts of extracellular miRNAs (Kosaka et al., 2010a). An independent group of authors further demonstrated that inhibition of nSMase does not alter intracellular miRNA levels but reduces miRNA in secreted exosomes (Kogure et al., 2011). While these data emphasize the importance of the MVBs and sphingomyelins for miRNA excretion, how exactly the selection and the loading of specific miRNA into exosomes occurs remains unknown. Finally, cell targeting has been hypothesized to be mediated by both exosomal surface proteins and receptors on the acceptor cells. The putative mechanisms of membrane vesicles uptake can be either direct membrane fusion or endocytosis (Thery et al., 2002; Cocucci et al., 2009; Simons and Raposo, 2009).

As it was mentioned before, some extracellular miRNA was co-purified with HDL from human blood (Vickers et al., 2011). HDL particles were able to deliver miRNAs to recipient cells and mediate direct targeting of mRNA reporters, while contrary to exosomes, cellular export of HDL associated miRNAs was negatively regulated by nSMase2. In addition, HDL mediated miRNA delivery was dependent on a cell surface HDL receptor SRBI, which binds HDL and mediates the uptake of cholesteryl ester from HDL. Because small RNAs can easily complex with zwitterionic liposomes it was hypothesized that HDL could simply bind to extracellular plasma miRNAs through divalent cation bridging (Vickers et al., 2011). This hypothesis, however, assumes the existence of naked mature miRNAs in the cell. Furthermore, targeting of mRNA by miRNA requires the latter to be associated with one of the AGO proteins. Importantly, neither formation of mature miRNAs nor their existence apart from AGO proteins has been found *in vivo*. It is, therefore, feasible that mature miRNAs in exosomes, MVs and HDL particles can be also bound to AGO proteins. Interestingly, recent evaluation of the HDL-bound miRNAs isolated from human blood revealed that the concentration of the most abundant HDL-bound miRNA miR-223 contributed to only 8% of the total miR-223 in the circulation (Wagner et al., 2013). Furthermore, no significant uptake of HDL-bound miRNAs was observed into endothelial cells, smooth muscle cells or peripheral blood mononuclear cells (Wagner et al., 2013).

## Extracellular miRNAs associated with microvesicles

Two different types of extracellular MVs described so far are shedding vesicles and exosomes. Exosomes are 30–100 nm in size, formed within the MVBs and released upon fusion of MVBs with the plasma membrane (Thery et al., 2002). Unlike exosomes, shedding vesicles are formed by outward budding and fission of the plasma membrane and can vary in size from 0.1 to 1 μm (Cocucci et al., 2009). Both types of MVs contain various proteins, mRNAs and miRNAs in a proportion depending on the cell from which they originate (Simons and Raposo, 2009; Muralidharan-Chari et al., 2010). Due to the similar size of exosomes and small shedding vesicles, it is impossible to completely separate them using differential ultracentrifugation or other physical methods. It has to be mentioned that most current reports describing isolation of MVs-associated extracellular miRNA rely on using solely ultracentrifugation. As a result, such experiments inevitably characterize miRNAs in a mixed population of two MVs types. However, researchers often refer to the miRNA isolated from ultra-centrifuged MVs to as “exosomal” miRNA. To our knowledge, there are no reports describing specifically “shedding vesicles” miRNA or specifically “exosomal” miRNA.

Exosomes can have many cell-type specific functions which were attributed predominantly to exosomal surface proteins. For example, Fas ligand located on the surface of tumor exosomes induces apoptosis in T lymphocytes (Abusamra et al., 2005). The biological function of the exosomal RNA *in vivo* remains questionable. However, numerous experiments performed on cultured cells have demonstrated that exosomal miRNAs can affect gene expression in the recipient cells and mediate a physiological response. A growing body of evidence that miRNAs can play a role in intercellular communication suggests the paracrine function of miRNAs which are packed in extracellular MVs (reviewed in Cortez et al., 2011; Chen et al., 2012; Turchinovich et al., 2012). The Internet-based database, ExoCarta (http://www.exocarta.org/), currently lists 463 miRNAs which were found in exosomes from various cells. In addition, the phenomenon of cell-cell communication via extracellular miRNAs has been shown in multiple cell culture models (Table 1).

**Table 1:** **Reports demonstrating cell–cell transfer of extracellular miRNA**.

**Donor cells**	**Acceptor cells**	**miRNA**	**Impact**	**References**
MC/9, HMC-1	MC/9 and HMC-1	Multiple miRNAs	Not investigated	Valadi et al., 2007
THP-1	HMEC-1 cells	Overexpressed miR-150	Reduction of miR-150 target c-Myb	Zhang et al., 2010b
			Increase in HMEC-1 cells migration	
EBV-infected B-cells	Dendritic cells, HeLa	Mature EBV-encoded miRNAs	Repression of EBV-miRNAs target CXCL11	Pegtel et al., 2010
COS-7, HEK 293	COS-7, HEK 293	Overexpressed luciferase siRNA	Repression of luciferase reporter gene	Kosaka et al., 2010a
COS-7	PC-3M (metastatic prostate cancer cells)	Overexpressed miR-146a	Decrease in proliferation	Kosaka et al., 2010a
			Repression of miR-146a target ROCK1	
Mesenchymal stem cells	Tubular epithelial cells (mTEC)	Multiple miRNAs	Repression of PTEN, cyclin D1, Bcl-2 proteins	Collino et al., 2010
Macrophages	SKBR3 and MDAMB-231 breast cancer cells	Endogenous miR-223	Reduction of miR-223 targeted Mef2c mRNA	
			Increased migration of SKBR3 and MDA-MB-231 cells	Yang et al., 2011
J77 T-cells, primary T-cells	Raji B cells (antigen presenting cells)	Overexpressed miR-335, endogenous miR-335, miR-92	Repression of miR-335 targeted 3′-UTR of SOX4 gene	Mittelbrunn et al., 2011
Hep3B	Hep3B	Endogenous miRNAs enriched in exosomes	Repression of putative miRNAs target TAK1	Kogure et al., 2011
Primary mesenchymal stromal cells	Primary astrocytes and neurons	Endogenous miR-133b	Increase in neurite outgrowth	Xin et al., 2012
Primary dendritic cells	Primary dendritic cells	Endogenous miR-451 and miR-148a	Repression of luciferase reporter	Montecalvo et al., 2012
HUVEC	Aortic smooth muscle cell	Endogenous miR-143/145	Repression of multiple miR-143/145 targets	Hergenreider et al., 2012
			Protection against atherosclerotic lesion formation	

Collino and co-authors incubated murine tubular epithelial cells (mTEC) with MSC-derived MVs and confirmed the transport of selected miRNAs by qRT-PCR (Collino et al., 2010). The abundance of extracellular miRNAs in acceptor cell increased progressively and correlated with the extent of MV internalization. Additionally, incubation of mTEC with MSC-derived MVs resulted in the reduction of proteins known to be targeted by some of the enriched miRNAs found in MVs including: PTEN (targeted by miR-21), cyclin D1 (targeted by miR-100, miR-99a, and miR-223) and Bcl-2 (targeted by miR-34, miR-181b, and miR-16).

Microvesicular miRNA from macrophages have been shown to enhance the invasiveness of breast cancer cells in culture (Yang et al., 2011). Specifically, macrophages activated by treatments with IL-4 secreted exosomes packed with miR-223 and were able to promote migration of SKBR3 and MDA-MB-231 breast cancer cells in a transwell invasion assay. Blocking miR-223 with antisense oligonucleotides prevented the observed increase of invasion capacity. In addition, (1) miR-223 targeted Mef2c mRNA level was reduced in the exosome-treated cells, and (2) the expression of β-catenin in the nucleus increased. Based on their observations, the authors suggested that miR-223 was transferred from macrophages to breast cancer cells via exosomes where it affected the Mef2c-β-catenin pathway leading to invasiveness of the breast cancer cells. For the first time it was suggested that prevention of the exosomal communication between macrophages and breast cancer cells may help preventing cancer metastasis and being potential target for cancer therapy (Yang et al., 2011).

The capacity of exosomal miRNA to facilitate viral infection was reported by Pegtel and co-authors. After infection of B-lymphoblastoid cells with Epstein-Barr virus (EBV) the viral-specific miRNAs (EBV-miRNAs) were secreted via exosomes and affected the expression of EBV-miRNA target gene CXCL11 in co-cultured non-infected cells (Pegtel et al., 2010). Viral miRNAs were present in both B-cell and non-B-cell fractions isolated from infected patients, while viral DNA was restricted to the circulating B-cell population. This indicated that viral miRNAs transfer from infected to non-infected cells also occurs *in vivo*.

Another evidence for functional cell-to-cell miRNA transfer was found during investigation of the immune synapse formation. Mittelbrunn and co-authors showed that exosomes of T, B, and dendritic immune cells contained different miRNA repertoires. Furthermore, miRNAs were transported from T cells to antigen presenting cells unidirectionally and this transport was antigen-driven (Mittelbrunn et al., 2011). In addition, transferred miRNAs could modulate gene expression in recipient cells. The exosomal miRNA-based communication between different dendritic cells has also been reported, resulting in the repression of target mRNAs of acceptor dendritic cells (Montecalvo et al., 2012).

MVs produced by THP1 monocyte/macrophage cells have been shown to deliver FITC-labeled exogenous miR-150 to HMEC-1 endothelial cells in culture (Zhang et al., 2010b). In addition, the delivery of miR-150 correlated with the reduction of a validated miR-150 target c-Myb and was accompanied with an increase in HMEC-1 cells migration. Treatment of HMEC-1 cells with specific miR-150 inhibitor abrogated the observed increase in migration. While the effect was observed in cultured cells over-expressing miR-150, it remains unknown whether extracellular levels of endogenous miR-150 in body fluids is high enough to significantly affect gene expression in targeted cells *in vivo*. However, the observation that plasma MVs isolated from atherosclerotic patients contained elevated levels of miR-150 ignited a hypothesis that secreted endogenous miR-150 may play a role in regulating endothelial cell migration.

Multipotent mesenchymal stromal cells are known to interact with brain parenchymal cells and promote their functional recovery. In the work of Xin et al., mesenchymal stromal cells exported miR-133b to the ipsilateral hemisphere. In addition, miR-133b was highly abundant in the primary cultures of neurons and astrocytes treated with exosome-enriched fraction released by mesenchymal stromal cells (Xin et al., 2012). Authors further showed that gap junction intercellular communication was important for the reported exosome-based miRNA transfer.

The elegant approach was used by Skog et al. to prove that exosomal RNA originating from glioblastoma tumor cells is taken up by recipient cells (Skog et al., 2008). The authors incorporated mRNA encoding luciferase reporter into exosomes and monitored luminescence of the recipient cells. Glioblastoma-derived MVs stimulated proliferation of a human glioma cell line enhancing further tumor progression. Besides, the authors demonstrated that serum MVs from glioblastoma patients contain mRNAs and miRNAs characteristic for gliomas and thus provide a potential diagnostic use.

Interestingly, the miRNAs from the let-7 family were found within the exosomes exported from the cultured metastatic gastric cancer cell line AZ-P7a but not from less metastatic cell lines (Ohshima et al., 2010). Because these miRNAs are known to be tumor-suppressive, the authors suggested that their elimination via exosomal export can maintain the oncogenic properties of the metastatic cells.

Hepatocellular carcinoma cells (HCC) have been shown to produce exosomes with specific mRNA, miRNA, and protein content (Kogure et al., 2011). The miRNAs highly enriched within HCC exosomes were predicted to target transforming growth factor β activated kinase-1 (TAK1), which contributes to local spread, intrahepatic metastases, or multifocal growth of this type of carcinoma cells (Kogure et al., 2011). Indeed, HCC-derived exosomes modulated TAK1 expression and enhanced transformed cell growth in recipient HCC in culture.

Another cancer-based model was based on human renal cancer stem cells. Grange and colleagues reported that a subset of tumor-initiating MSCs from human renal cell carcinoma released MVs which triggered angiogenesis and promoted the formation of a pre-metastatic niche. Importantly, cancer stem cell MVs contained miRNAs implicated in tumor progression and metastases, and conferred an angiogenic phenotype to normal human endothelial cells, stimulating their growth and vessel formation (Grange et al., 2011). However, it remains uninvestigated whether the miRNAs were responsible for the observed physiological impact.

Hergenreider and co-authors have found that extracellular vesicles mediate miRNA transfer from human endothelial cells to smooth muscle cell *in vitro*. Specifically, membrane vesicles secreted by shear-stressed cultured endothelial cells were enriched with miR-143/145 and modulated gene expression in co-cultured smooth muscle cells (Hergenreider et al., 2012). Moreover, miR-143/145-containing vesicles inhibited atherosclerotic lesion formation in the aorta in a mouse model suggesting a potential therapy against atherosclerosis.

## Extracellular miRNAs associated with other carriers

The products of cell apoptosis (or programmed cell death) are apoptotic bodies (AB) 1–2 μm in size (Kerr et al., 1972; Hengartner, 2000; Hristov et al., 2004). Together with exosomes and MVs, some researchers consider ABs as carriers of cell–cell communication information. Thus, both viral and chromosomal DNA can be transferred between somatic cells by uptake of the apoptotic bodies (Holmgren et al., 1999; Bergsmedh et al., 2001). Zernecke et al. has shown that ABs inhibit atherosclerosis progression when injected into the blood circulation. The authors also proposed that miR-126 encapsulated into ABs may be responsible for this protective effect via induction of the chemokine CXCL12 expression. Indeed ABs contained miR-126 and delivered miR-126 to recipient vascular cells (Zernecke et al., 2009). Furthermore, injections of miR-126 containing apoptotic bodies reduced manifestations of atherosclerosis in mice, while apoptotic bodies isolated from miR-126-deficient animals did not have such an effect. The protective effect was accompanied by elevated expression of CXCL12 in the carotid arteries. It has to be mentioned that in their experimental model the authors describe incubation of carotid arteries with relatively high concentrations of ABs *in vitro*. It remains to be tested whether physiological levels of ABs would affect gene expression in a similar manner.

A single report demonstrating that Hepatitis B subviral surface antigen particles (HBsAg) circulating in the Hepatitis B infected carriers contain both hepatocellular miRNAs and the AGO2 associated protein (Novellino et al., 2012). Interestingly, HBsAg associated miRNAs were liver-specific (miR-27a, miR-30b, miR-122, miR-126, and miR-145) as well as immune regulatory (miR-106b and miR-223). Computationally predicted target genes of HBsAg-associated miRNAs included molecular pathways of host-pathogen interactions.

Solely AGO protein-associated miRNA represents by far the largest class of extracellular miRNA (Arroyo et al., 2011; Turchinovich et al., 2011; Turchinovich and Burwinkel, 2012). It was hypothesized that the AGO-ribonucleoprotein complexes are passively released by all cells after either necrotic or apoptotic death and remain stable in the extracellular space due to the high stability of the AGO proteins (Turchinovich et al., 2011; Turchinovich and Burwinkel, 2012). However, it cannot be completely excluded that certain cell membrane-associated channels or receptors mediate specific release of some AGO-miRNA complexes. Interestingly, in *C. elegans*, cellular uptake of dsRNA is mediated by a transmembane channel protein SID-1 (Feinberg and Hunter, 2003). In addition, SID-1 is capable of importing synthetic miRNA precursors and long hairpin molecules into the cell (Shih and Hunter, 2011). While the mammalian homologs of SID proteins do exist, it remains unclear whether they can uptake RNA from extracellular fluids (Duxbury et al., 2005; Wolfrum et al., 2007). Furthermore, it remains to be evaluated whether AGO-bound single stranded mature miRNA can be recognized by SID proteins in a similar manner as “naked” double stranded RNA.

Amazingly, two recent research reports suggest that extracellular miRNA may work in non-canonical ways. Specifically, either dead cell-released or exosomes secreted miRNAs can act as signaling molecules to mediate intercellular communication via binding to extracellular or intracellular Toll-like receptors (TLRs) (Fabbri et al., 2012; Lehmann et al., 2012). TLRs are a family of innate immune system receptors which recognize various molecular patterns of microbial pathogens and induce antimicrobial immune responses (Takeda et al., 2003; Blasius and Beutler, 2010). In 2001, Alexopoulou and co-authors first showed that dsRNA binds to mammalian TLR-3, consequently leading to the activation of NF-kappaB and the production of type I interferon response (Alexopoulou et al., 2001). Later, Kleinman and co-authors reported that cell surface TLR-3 mediates extracellular siRNA-induced inhibition of angiogenesis independently of siRNA sequence (Kleinman et al., 2008). The intracellular TLRs located within endolysosomal compartments can also bind both double stranded and single stranded nucleic acids derived from viruses and bacteria (Heil et al., 2004). Among the major effects of the activation of intracellular TLRs is the induction of cytokines essential for innate immune responses. In their work, Fabbri et al. showed that miR-21 and miR-29a secreted by tumor cells are capable of binding to murine TLR-7 and human TLR-8 in immune cells, triggering secretion of prometastatic inflammatory cytokines that ultimately may lead to tumor growth and metastasis (Fabbri et al., 2012). The authors also concluded that extracellular miRNAs could function as key regulators of the tumor microenvironment by acting as paracrine agonists of TLRs (Fabbri et al., 2012). The recent report of Lehmann and colleagues provided further evidence in favor of the unconventional role for the extracellular miRNAs (Lehmann et al., 2012). Intrathecal injection of extracellular let-7b into the cerebrospinal fluid of wild-type mice, but not TLR7 knockouts, resulted in activation of microglia/macrophages and neurodegeneration. Furthermore, susceptibility to let-7-induced toxicity was restored in neurons transfected with TLR7 by intrauterine electroporation of Tlr7^−/−^ embryos. The authors also observed that: (1) dying neurons released let-7b *in vitro*; and (2) levels of let-7b were increased in CSF from patients with Alzheimer's disease (Lehmann et al., 2012). These results suggest that extracellular miRNAs can function as signaling via TLR-7 pathway and contribute to the spread of CNS damage.

In 2011, Vickers and colleagues reported that HDL complexes isolated from human blood plasma contain miRNA and could transmit this miRNA into other cells (Vickers et al., 2011). To examine whether miRNAs carried by HDL can alter gene expression in distant cells, HDL were isolated from hypercholesterolemia patients and healthy subjects. Treatment of human hepatocytes in culture with HDL derived from hypercholesterolemia subjects significantly increased the level of miR-105 in these cells, whereas HDL from healthy controls had no such effect. Further microarray analysis revealed that HDL from hypercholesterolemia patients induced profound alterations in mRNA expression including downregulation of multiple putative targets of miR-105 in cultured hepatocytes. Contrary to exosomes, cellular export of HDL-associated miRNAs was negatively regulated by nSMase2. In addition, HDL mediated miRNA delivery was dependent on a cell surface HDL receptor SRBI, which binds HDL and mediates the uptake of cholesterylester from HDL.

## Conclusion and future perspective of the field

Despite a number of fascinating examples of intercellular communication via miRNA between cells in culture, the physiological significance of such paracrine or endocrine impact in the body is challenged by the fact that the vast majority of the extracellular miRNA are present in membrane-vesicle-free AGO protein-associated form. Furthermore, the concentration of miRNA in the biological fluids is drastically lower than in the surrounding cells and might be below the threshold for triggering any significant physiological effect *in vivo* (Turchinovich et al., 2011; Williams et al., 2013). Finally, so far extracellular miRNA trafficking was consistently shown: (1) only in cultured cells; and (2) only for several miRNAs.

In their recent report Tuschl group argues against a hormone-like effect of extracellular miRNA in the blood (Williams et al., 2013). Deep sequencing experiments revealed that the concentration of total miRNA in the plasma is within 100 fM range, and the concentration of any individual miRNA is only a fraction of this number. However, even the lowest level trace hormones in the blood are present at least in the picomolar concentration range. The action of hormones implies receptor-binding and multimillion amplification of the transmitted signal within the cell. Unlike hormones miRNAs require intracellular levels of greater than 1000 copies per cell to exert measureable activity on their mRNA targets (Williams et al., 2013). Based on these calculations the authors concluded that it is unlikely that miRNAs can function as hormones unless they bind to a sensitive miRNA receptor (Williams et al., 2013).

The paracrine mode of cell–cell signaling for extracellular miRNA appears to be more feasible. Indeed most, if not all, current reports describe evidence of rather short distance communication of cells via extracellular miRNA. Unlike average miRNA levels in a biological fluid, the local concentrations of extracellular miRNAs could suffice to secure the delivery of physiologically relevant amounts of miRNA from donor to neighboring acceptor cell. Recent evidence of interaction of miRNA with TLRs provided additional complexities to distinguish sequence-specific effects of extracellular miRNA on the targeted mRNAs expression in acceptor cells and non-specific response of the innate immune system (Fabbri et al., 2012; Lehmann et al., 2012).

Despite the fact that extracellular miRNA circulating in bio-fluids has many properties of promising biomarkers for various pathological conditions, the concept of miRNA mediated cell-cell signaling in vertebrates requires further validation. Among the central questions to be answered remains: (1) whether solely protein-bound extracellular miRNA can penetrate through the cell membrane and if so, which mechanisms are responsible; (2) whether concentrations of MVs-associated miRNAs are above the physiological limit to mediate any significant para- or endocrine signaling *in vivo*; (3) what are the mechanisms of selective export of miRNAs into extracellular space; (4) how many miRNAs out of the total extracellular pool participate in cell–cell signaling.

### Conflict of interest statement

The authors declare that the research was conducted in the absence of any commercial or financial relationships that could be construed as a potential conflict of interest.
